# The evolution of knowledge within and across fields in modern physics

**DOI:** 10.1038/s41598-020-68774-w

**Published:** 2020-07-21

**Authors:** Ye Sun, Vito Latora

**Affiliations:** 1 School of Mathematical Sciences, Queen Mary University of London, London, E1 4NS UK; 2 Dipartimento di Fisica ed Astronomia, Università di Catania and INFN, 95123 Catania, Italy; 3 The Alan Turing Institute, The British Library, London, NW1 2DB UK; 4 Complexity Science Hub Vienna (CSHV), Vienna, Austria

**Keywords:** Applied physics, Applied mathematics

## Abstract

The exchange of knowledge across different areas and disciplines plays a key role in the process of knowledge creation, and can stimulate innovation and the emergence of new fields. We develop here a quantitative framework to extract significant dependencies among scientific disciplines and turn them into a time-varying network whose nodes are the different fields, while the weighted links represent the flow of knowledge from one field to another at a given period of time. Drawing on a comprehensive data set on scientific production in modern physics and on the patterns of citations between articles published in the various fields in the last 30 years, we are then able to map, over time, how the ideas developed in a given field in a certain time period have influenced later discoveries in the same field or in other fields. The analysis of knowledge flows internal to each field displays a remarkable variety of temporal behaviours, with some fields of physics showing to be more self-referential than others. The temporal networks of knowledge exchanges across fields reveal cases of one field continuously absorbing knowledge from another field in the entire observed period, pairs of fields mutually influencing each other, but also cases of evolution from absorbing to mutual or even to back-nurture behaviors.

## Introduction

Knowledge creation and knowledge sharing go hand in hand. Knowledge is in fact created through combination and integration of different concepts, and can benefits from social interactions and interdisciplinary collaborations. Recent works have explored from many angles how knowledge flows across scholars^1–5^, institutions^6–9^ and disciplines^10–12^. In particular, it has been shown that knowledge exchange across fields can influence the evolution of culture and language^13,14^, strengthen multi-faceted cooperation^3,15^, and drive the innovation and development of science^16–19^. Research publications are one of the primary channels of communication for the exchange and spreading of knowledge in science^20^. By publishing their own articles and citing works by their peers, researchers continuously contribute to the processes of knowledge creation, knowledge sharing and knowledge acquisition^21^, thereby promoting the advancement of science. The presence of a citation between two research articles often denotes a certain transfer of knowledge from the cited article to the citing articles. It is therefore natural to use citations between articles published in different scientific fields to investigate the flow of knowledge across different domains of science. Despite some works in this direction have already started elucidating the main mechanisms of knowledge sharing and diffusion^22–24^, a systematic study on how knowledge evolves in time^25^ and of the complex interactions and influences between different fields^26^ is still lacking.

In this article, we propose a novel framework to detect and quantify relevant transfers of knowledge across disciplines and between different time periods. One of the outcomes of the method is the construction of a time-varying network mapping the structure of knowledge and the relations between disciplines. In particular, we present an application to study the evolution of scientific knowledge in modern physics, namely to investigate how influences from one field of physics to another have evolved over time in the last 30 years. Building on bibliographic information of over 430,000 articles published by the American Physical Society (APS) between 1985 and 2015, and making use of the highest-level Physics and Astronomy Classification Scheme (PACS) codes, which indicate the fields of physics an article belongs to, we construct a temporal network where the nodes represent the fields of modern physics and the directed links denote the presence of a significant dependence of a field on another. Such a network is changing over time and, as we will show below, its analysis by the methods of network science^27,28^ is able to reveal essential properties of how knowledge is exchanged among fields and over different time periods. We have found that, overall, knowledge flows have become increasingly homogeneous over the last years, indicating the important role of interdisciplinary research^10,11,29–31^. In spite of this, some typical patterns of influence, such as cases of one field absorbing knowledge from another field, or two fields mutually influencing each other, clearly emerge at the microscopic scale. Our findings provide insights into the basic mechanisms of knowledge exchange in science, and can turn very useful to understand the dynamics of scientific production and the growth of novelties in scientific domains^32–34^.

## Results

### The fields of modern physics

An overall insight into the main fields of modern physics can be obtained by a basic analysis of the characteristics of the APS data sets. Considered at their highest level, PACS codes divide modern physics into ten major fields (see Table 1). A measure of the relevance of each field can then be derived from the volume of papers published in each field. Since each paper can be listed with multiple PACS codes we assign it to multiple fields. We therefore consider each paper as one unit of knowledge and define the field composition of the paper as the relative frequency of its PACS codes. For instance, if a paper is listed with three PACS codes ${\textit{89.75.-k}}$, ${\textit{81.05.-t}}$ and ${\textit{05.45.-a}}$, we assign two-thirds of this paper to *Interdisciplinary Physics* (PACS 80), and the remaining one-third to *General Physics* (PACS 00). The total number of papers $N_{paper}$ associated to each field over the entire time period of 31 years is reported in Table 1. One can see that the three largest fields are *Condensed Matter* (PACS 60 and 70) and *General Physics* (PACS 00), capturing $57\%$ of the entire publications in APS journals. *GPE* is the smallest field, with only 8325 papers, which is roughly one-fifteenth the size of the largest field *CM*2. To quantify and compare the growth rate of each field, the average yearly change in the number of papers $\Delta N_{paper}$ is also reported in Table 1. Consistently with the rankings based on field sizes, *GEN* and *CM*2 also exhibit the highest growth rate, larger than 100 papers per year, while *GPE* shows the slowest increase, with only 5 papers per year on average. On the contrary, *CM*2, the third-largest field by size, is ranked fourth from the bottom according to the average growth $\Delta N_{paper}$. An opposite trend is observed for *Interdisciplinary Physics* and *Astrophysics*, which respectively take fifth and sixth place according to their growth rates, although their field sizes are ranked eighth and ninth among these fields, reflecting their rapid development during the observing period.

We found that $91\%$ of the papers have more than one PACS code, with $36\%$ of them reporting PACS codes which are at least from two different fields. To quantify the level of interdisciplinarity of a given field, we have collected all the papers with at least one PACS code from that field, and then calculated the proportion *J* of these papers which are also classified by at least one PACS code from other fields. The results in Table 1 show that *Interdisciplinary Physics* is the field with the largest value of *J*: almost $90\%$ of the Interdisciplinary Physics papers are also classified by PACS codes from other fields of physics. This result is consistent with the expectation that interdisciplinary research combines knowledge from various disciplines. Instead, papers in the fields of *Nuclear*, *Particles* and *Condensed matter* 2 physics are more likely to use PACS codes from their own fields. Summing up, the above analyses indicate that the differences between fields of physics are remarkable, either in terms of the size and growth of the fields, and in terms of their interactions with other fields.

**Table 1:** The ten fields of modern physics.

PACS	Abbreviation	Field information	$N_{paper}$	$\Delta N_{paper}$	*J*
00	GEN	General physics	66,909	115	0.76
10	EPF	The physics of elementary particles and fields	46,722	56	0.44
20	NUC	Nuclear physics	29,120	17	0.42
30	ATM	Atomic and molecular physics	28,929	10	0.64
40	EOA	Electromagnetism, optics, acoustics, heat transfer, classical mechanics, fluid dynamics	35,425	58	0.79
50	GPE	Physics of gases, plasmas, electric discharges	8,325	5	0.62
60	CM1	Condensed matter: structural, mechanical and thermal properties	53,287	21	0.78
70	CM2	Condensed matter: electronic structure, electrical, magnetic, optical properties	127,319	106	0.46
80	IPR	Interdisciplinary physics and related areas of science and technology	24,346	51	0.89
90	GAA	Geophysics, astronomy, astrophysics	15,319	34	0.78

PACS codes and names of the main fields of physics as defined at the highest level of the APS hierarchical classification scheme. $N_{paper}$ represents the total number of papers published in each field in the period between 1985 and 2015. $\Delta N_{paper}$ denotes the average yearly increase in the number of papers in each field, which is calculated as the slope coefficient in a linear regression of $N_{paper}$ versus time. Among all the papers in an observed field, *J* indicates the proportion of these papers that are also classified with at least one PACS code from other fields.

### The knowledge flow network

Interactions among scientific fields can be better characterized by making use of scientific citations. A published article in a scientific field citing articles of another field implies that the cited field reflects a piece of previously existing knowledge that the citing field builds upon. And this, in turn, indicates a flow of knowledge from the cited field to the citing field. Hence, we can construct a network of knowledge flow across fields by analyzing the pattern of citations among papers of different fields. The nodes of such a network represent the ten fields of modern physics as indicated by the PACS codes, while the directed links between fields denote the flows of knowledge from one area of physics to another.

**Figure 1:**
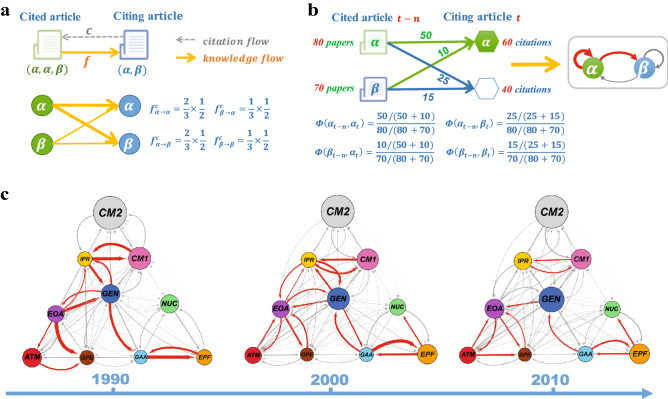
The knowledge flow network and its time evolution. (**a**) Illustration of how a citation between two papers is translated into a contribution to the knowledge flow between the two corresponding fields. (**b**) Construction of a weighted network of knowledge flow based on the significance of each link. (**c**) The knowledge flow network among different fields in physics in years 1990, 2000 and 2010. Node sizes are proportional to the number of papers published in each field and given year, and the line widths correspond to the weights of knowledge flows between two fields. The links with weights larger than 1 are selected with red color. The arrow represents the direction of knowledge flows.

Specifically, for a given citation *c* there will be a transfer of knowledge from each PACS code in the cited reference to all the PACS codes in the citing article. We hence indicate as $f^{c}_{\alpha \rightarrow \beta }$ the volume of knowledge flow from field $\alpha$ to field $\beta$ due to citation *c*. As shown in Fig. 1a, this is calculated as the product of the proportion of PACS codes from field $\beta$ in the citing article times the proportion of PACS codes from field $\alpha$ in the cited article. This ensures the normalization $\sum _{\alpha }\sum _{\beta }{f}^{c}_{\alpha \rightarrow \beta }=1$ for each citation *c*, meaning that each citation contributes a unit of knowledge transfer that is then split to the different fields. For instance, in Fig. 1a, two of the three PACS codes of the cited article belong to field $\alpha$, while one over two of the PACS codes in the citing article is from field $\beta$. Consequently, we assume that the volume of knowledge flowing from field $\alpha$ to field $\beta$, due to citation *c*, is $f^{c}_{\alpha \rightarrow \beta }=2/3 \times 1/2$. Similarly, we can calculate the quantities $f^{c}_{\alpha \rightarrow \alpha }$, $f^{c}_{\beta \rightarrow \alpha }$ and $f^c_{\beta \rightarrow \beta }$. In order to characterize the flow of knowledge across fields and to study its evolution over the years, we construct yearly aggregated networks by selecting different pairs of years for citing and cited articles respectively. This is done by analyzing all the citations from papers published in a given year *t* to papers published in year $t-n$, and defining the total volume of knowledge flowing from field $\alpha$ to field $\beta$ as:

1 $$F_{\alpha \rightarrow \beta }^{t-n \rightarrow t} = \sum _{\mathrm{cit}} f^{c}_{\alpha \rightarrow \beta }$$

where the sum runs over all citations *c* from papers published in field $\beta$ in year *t* to papers published in field $\alpha$ in year $t-n$. Notice that *n* is a tunable parameter, denoting the relative age of cited papers with respect to the citing year *t*. Having the possibility to vary both *t* and *n* allows to take into account that the probability of a citation is influenced by: (1) the relative age of the two papers^35^, and by (2) the number of papers published in the cited year $t-n$. The quantities $F_{\alpha \rightarrow \beta }^{t-n \rightarrow t}$ are, however, affected by field-specific characteristics and publishing conventions, such as typical field sizes and time-varying growth rates, which, as shown in Table 1, may vary a lot from field to field. Some other influencing factors that are not discussed here might exist, such as the difference of reference list length across fields, which could be considered in more detailed study. Hence, an increase of $F_{\alpha \rightarrow \beta }^{t-n \rightarrow t}$ over time does not automatically reflect a closer relation between fields $\alpha$ and $\beta$, as it can only be due to a rapid growth in the number of publications in these two fields. In order to account for this, we define the statistical significance $\phi (\alpha _{t-n},\beta _{t})$, which quantifies how the observed knowledge flow $F_{\alpha \rightarrow \beta }^{t-n \rightarrow t}$ exceeds the flow expected in a opportunely chosen null model (see the “Methods” section). Figure 1b illustrates an example of how to calculate the quantities $\phi (\alpha _{t-n},\beta _{t})$ in Eq. (5). Suppose, for instance, field $\alpha$ in year $t-n$ provides a total of 75 units of knowledge to all the fields in year *t*, with field $\alpha$ itself receiving 50 of these 75 units, and $\beta$ getting 25, i.e. $F_{\alpha \rightarrow \alpha }^{t-n \rightarrow t}=50$ and $F_{\alpha \rightarrow \beta }^{t-n \rightarrow t}=25$. Analogously we assume field $\beta$ in year $t-n$ provides a total of 25 units to year *t*, 15 to field $\beta$ itself and 10 to $\alpha$. Now, the marginal probability that the citing field is $\beta$ can be obtained as $Pr(X_{t}^{citing}=\beta )=\sum _{\alpha } F_{\alpha \rightarrow \beta }^{t-n \rightarrow t}/\sum _{\alpha ,\beta } F_{\alpha \rightarrow \beta }^{t-n \rightarrow t}=(25+15)/(75+25)$, while $Pr(Y_{t-n}^{cited}=\alpha | X_{t}^{citing}=\beta )= F_{\alpha \rightarrow \beta }^{t-n \rightarrow t}/\sum _{\alpha } F_{\alpha \rightarrow \beta }^{t-n \rightarrow t} = 25/(25+15)$. We therefore have $P(\alpha _{t-n}, \beta _{t}) = 25/40 \cdot 40/100$. Such a probability needs to be compared to that of a null model in which $P^{\mathrm{rand}}(\alpha _{t-n}, \beta _{t})=Pr(Y_{t-n}^{cited}=\alpha ) \cdot Pr(X_{t}^{citing}=\beta )= 80/150 \cdot 40/100$, since the probability $Pr(Y_{t-n}^{cited}=\alpha )$ that the cited paper in year $t-n$ is in field $\alpha$ is equal to $80/(80+70)$. Finally, the ratio $\phi (\alpha _{t-n},\beta _{t})$ in Eq. (5) is equal to $25/40 \cdot 150/80$.

Analogously, we can calculate the statistical significance of all the other flows reported in Fig. 1b. Such quantities allow to capture the intrinsic variation of knowledge flows among fields and also to compare different pair of fields. Finally, to obtain the weights of knowledge flows in year *t* from a cited time window $\Delta t'$, we define the flow weights $w_{\alpha \rightarrow \beta }^{\Delta t' \rightarrow t}$ for each couple of cited field $\alpha$ and citing field $\beta$ as:

2 $$\begin{aligned} w_{\alpha \rightarrow \beta }^{\Delta t' \rightarrow t}= \frac{1}{\vert \Delta t' \vert }\sum _{n \in \Delta t'}{\phi (\alpha _{t-n}, \beta _{t})} \end{aligned}$$

where $\vert \Delta t' \vert$ is the length of the time window. Let $\Delta t'=[1, 5]$ and $\vert \Delta t' \vert =5$, then one can construct the significant knowledge flow network in each year *t* from the previous 5 years. Furthermore, for each given source period $\Delta t'$, one can also investigate the knowledge flows within an observing period $\Delta t$:

3 $$\begin{aligned} w_{\alpha \rightarrow \beta }^{\Delta t' \rightarrow \Delta t}= \frac{1}{\vert \Delta t \vert }\sum _{t \in \Delta t} w_{\alpha \rightarrow \beta }^{\Delta t' \rightarrow t} \end{aligned}$$

For example, let $\vert \Delta t \vert =5$, we can divide the entire time period into five observing time windows, namely [1990, 1994], [1995, 1999], [2000, 2004], [2005, 2009] and [2010, 2014]. The weight of each link in the network reflects how significant the knowledge flows between two related fields. This quantitative framework enable us to investigate the evolution of knowledge flows in two time dimensions: (1) for each given observing period $\Delta t$, the weights of knowledge flows from different time interval $\Delta t'$ can be observed; and also (2) for each fixed $\Delta t'$, the weights of knowledge flows within different observing period $\Delta t$ can be compared. Although in this paper we have studied knowledge flows across the ten major fields of physics, we believe that our framework can also give important information when applied to investigate knowledge transfer among subfields at any possible level of hierarchy.

### Temporal analysis of knowledge flow networks

We first investigate how the overall properties of the knowledge flow networks have changed over time. Specifically we have evaluated, for each year, the flows of knowledge from the previous 5 years, i.e we have fixed $\Delta t'=[1,5]$ and $\vert \Delta t \vert =1$ in our framework. To better visualize the temporal changes, the whole knowledge flow networks obtained for the 3  years 1990, 2000 and 2010 are reported in Fig. 1c. Links representing a significant flow of knowledge ($w_{\alpha \rightarrow \beta }^{\Delta t' \rightarrow t}>1$) are shown in red color.

**Figure 2:**
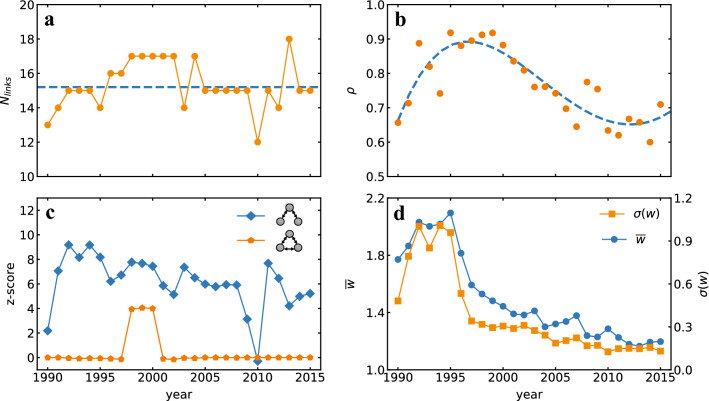
Temporal analysis of knowledge flow networks. (**a**) The number of significant links in the knowledge flow network is shown as a function of the year together with its time average, reported as a dashed gray line. (**b**) Network reciprocity measuring the proportion of bidirectional links, shows a pattern with a peak around year 1998. The top $50\%$ of bidirectional links with the largest sum of mutual weights were considered in the computation of the reciprocity. (**c**) The Z-score of two types of three-node motifs is reported as a function of time. (**d**) Mean and standard deviation of the weights of significant links gradually decrease over time, indicating that the knowledge flows between fields are tending more towards random expectations.

The first thing to notice is that the number of significant links is roughly constant over the years, as also illustrated in Fig. 2a. In addition to this, we observe that more links are reciprocated in 2000 with respect to years 1990 and 2010, which suggests that situation in which couples of fields mutually influence each other are more common 2000. To further examine this, we have computed the network reciprocity (see “Methods” section) for each year. The results reported in Fig. 2b indicate that the value of the reciprocity $\rho$ has increased in the first few years, reached a peak around 1998, and then has begun to decrease in the following years. This has lead us to conclude that the highest levels of mutuality in knowledge transfer among different fields of physics have been experienced between 1995 and 2000.

We have then extracted the typical patterns of knowledge transfer in the network. For this reason, we have focused on the statistically significant three-node motifs in the knowledge flow networks^36^, i.e. the directed connected subgraphs of three nodes that appear in the network more often than they would occur by chance. Figure 2c illustrates the Z-scores (see the “Methods” section) of two relevant three-node motifs over the years. One can see that the subgraph represented by bi-directed paths (diamond symbol) is the most significant motif throughout the whole time period, with a Z-score on average equal to about 6. Furthermore, complete subgraphs of three nodes, corresponding to three mutually connected fields of physics, are only statistically significant in the period from 1998 to 2000, where the complete subgraph *GEN*, *EOA* and *IPR* appears. Notice that this period also corresponds to the time period of high reciprocity in Fig. 2b.

In addition, Fig. 1c indicates that there are fewer links with large weights in year 2010 than in 1990 and 2000. To further investigate this trend, Fig. 2d reports mean $\overline{w}$ and standard deviation $\sigma _w$ of the weights of significant links between 1990 and 2016. We find that both the values of $\overline{w}$ and $\sigma _{w}$ gradually decrease overtime and eventually stabilize to values slightly above 1 and 0 respectively. This indicates that the exchange of knowledge across domains has increasingly become more homogeneous with respect to the beginning of 1980s, when each field only absorbed knowledge from a handful of close domains. From a different perspective, this also reflects a rise of the interdisciplinary character of research in physics.

### Internal knowledge flows

**Figure 3:**
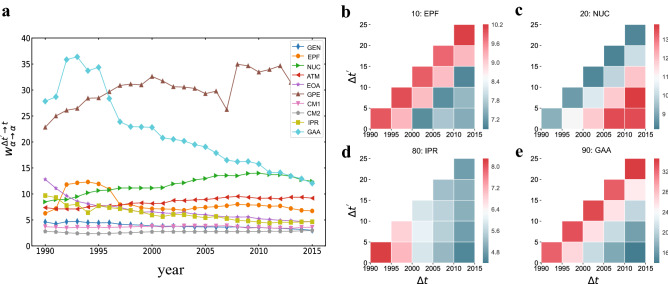
Evolution of knowledge flows within a field. (**a**) For each field $\alpha$ and each year *t*, we plot the internal knowledge flow $w_{\alpha \rightarrow \alpha }^{\Delta t' \rightarrow t}$ from a window of the previous 5 years to *t*. In (**b**)–(**e**) we show the evolution of internal flows for four specific fields in two-dimensional plots $\Delta t$, $\Delta t'$. By comparing the change in each row, we find that field *NUC* shows an increasingly high degree of self-reference over time, while, conversely, *IPR* and *GAA* tend to become less and less self-dependent. Focusing on the variation in each column, we can examine the effect of reference age on the significance of internal knowledge transfer. The lengths of each citing period $\Delta t$ and cited period $\Delta t'$ in (**b**)–(**e**) are both equal to 5 years.

The weights of the internal flows $w_{\alpha \rightarrow \alpha }^{\Delta t' \rightarrow t}$ from a field $\alpha$ to itself are an indication of the degree of self-dependence of the research field. To investigate the evolution of the internal knowledge flows, we have computed, for each of the ten fields of physics, the weights of the internal flows in every observing year *t* (between 1990 and 2015) from each of the previous 5 years, namely adopting a cited time window $\Delta t'=[1,5]$. Figure 3a indicates that the internal knowledge flows are significant ($w_{\alpha \rightarrow \alpha }^{\Delta t' \rightarrow t}>1$) for all ten fields over the whole 21-year period of time, although the temporal trends can vary from field to field. The two fields with the largest variations are *GAA* and *GPE*. Field *GAA* exhibits a remarkable decrease in the degree of self-reference after 1993, indicating that in this field the internal transfer of knowledge has become less and less significant over time. Conversely, field *GPE* shows an increasing trend and becomes the most self-referential field after 1995. Other fields exhibit decreasing (*EOA* and *IPR*) or increasing (*NUC* and *ATM*) patterns, while the contribution of internal flows are relatively low and keeps nearly constant for fields such as *GEN*, *CM1* and *CM2*.

In Fig. 3b–e we focus on the evolution of internal flows of the four fields *EPF*, *NUC*, *IPR* and *GAA*. In particular, we perform a two-dimensional analysis in which we change the positions of both the observing time windows $\Delta t$ and the source time window $\Delta t'$. We consider the case where the lengths of the two time windows is the same and is equal to 5 years. The colours in Fig. 3b–e represent the values of internal knowledge flows $w_{\alpha \rightarrow \alpha }^{\Delta t' \rightarrow \Delta t}$. By looking at the variations of colours in each row we find that field *NUC* shows an increasingly high degree of self-reference over time, while *IPR* and *GAA* tend to lower their degree of internal flows, which is consistent with the results in Fig. 3a.

By looking at the variation of colours over each column of Fig. 3b–e we can instead investigate the influence of reference’s age on the internal flows of knowledge. One can see that fields such as *NUC* and *IPR* show a decreasing trend from most recent times to the past, in agreement with previous studies stating that the likelihood of a paper being discovered significantly decreases with the papers’ age^37^. By contrast, we observe an unexpected and very clear pattern for *EPF* and *GAA*, since both fields exhibit a maximum of the values along the anti-diagonal line. Notice that each square along the anti-diagonal line represents the same cited time window, namely a time window of 5 years before the period [1990, 1994]. This may be due to important discoveries and the publication of pioneering research works in the fields *EPF* and *GAA* during the period [1985, 1990] which would clearly increase the probability for researchers in the field to cite, in the following years, papers published in that period. A possible explanation can be for instance the rapid development in the period [1985, 1989] of the new research area “*astroparticle physics*”, emerging at the intersection of particle physics, astronomy and astrophysics^38^, and which mainly combines the knowledge from fields *EPF* and *GAA*. As an evidence of this rapid development, notice that even a new journal named “Astroparticle Physics” was established in 1992. Moreover, the fact that the weights of the internal flows in *GAA* are nearly three times larger than those in *EPF*, can be due to the Hubble Space Telescope, one of the major scientific breakthroughs in field *GAA*. The telescope is one of the largest and most productive scientific research tool for astronomy, and it was indeed launched in 1990 (within the period of interest in the anti-diagonal line), greatly promoting the development of astronomy in *GAA*. We have further examined the evolution of internal flows for the remaining six fields and found similar patterns (see Supplementary Information (SI)).

### The evolution of knowledge flows across fields

**Figure 4:**
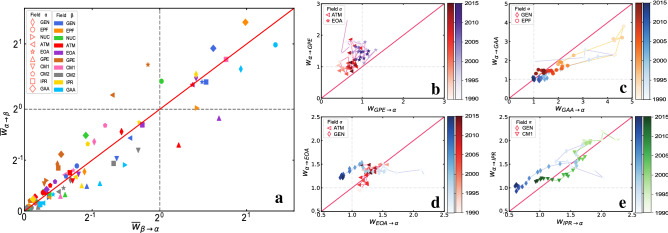
Evolution of knowledge across fields. (**a**) For each pair of fields, we plot the average flows of knowledge in either direction, averaged over the entire observation period of 26 years. (**b**)–(**e**) report some of the typical patterns of temporal evolution we have observed over the years. Symbol color (from light to dark) indicates the years from 1990 to 2015, while the lines join consecutive years to help following the trajectories. The bisector red line corresponds to the case of perfectly symmetric knowledge flows between the two fields. (**b**) “Absorbing mode”: field *GPE* has been absorbing knowledge from fields *ATM* and *EOA* throughout the entire time period. (**c**) “From absorbing to mutual mode”: *GEN* and *EPF* have initially absorbed more knowledge from field *GAA* and then tend to a balanced case in which they absorb from *GAA* the same knowledge they provide to it. (**d**) “Back-nurture mode”: while during the first few years *GEN* has absorbed more knowledge from field *EOA* than it has contributed to, at a later stage the situation is inverted. (**e**) “Mutual mode”: fields *IPR* and *CM1* tend to share knowledge in a symmetric way over the whole period.

Examining how the discoveries in a field have contributed to a different field of physics is even more important than studying the flows of knowledge within a given field. In order to get an overall picture of the existing influences across different fields of modern physics, we report in Fig. 4a the average weights of knowledge flows between each couple of fields over the whole period under study. To highlight the mutual exchange of flows, the results are shown in a ($\overline{w}_{\alpha \rightarrow \beta }$)-($\overline{w}_{\beta \rightarrow \alpha }$) plane. Each point refers to a pair of fields, and the distance from the position of the point to the bisector (red line) measures the level of asymmetry in the exchange of knowledge between the two fields. We notice that most of the points are concentrated around the bisector, especially those points in the lower-left corner corresponding to pairs of fields with small significance weights. However, there are also points far from the line, such as the point corresponding to the pair *GPE* and *ATM* (red up-triangle in the lower right of the panel), indicating asymmetric transfers of knowledge between two fields.

To investigate the temporal evolution in the exchange of knowledge between two fields in Fig. 4b–e, we have considered the same types of plots over time. In such a case, each pair of fields corresponds to a trajectory joining the points corresponding to the different years from 1990 to 2015. The colors of symbols from light to dark indicates the years from past to the most recent. Although the significance of the links in general decreases over time, the temporal patterns can vary from one pair of fields to another. The four panels illustrate the four major classes of behaviour (modes) we have found, namely: absorbing, absorbing to mutual, back-nurture and mutual mode. Absorbing mode can be seen in field *GPE*, which has absorbed more knowledge from fields *ATM* and *EOA* throughout the whole period under study (Fig. 4b). Fields *GEN* and *EPF* show a similar behavior as *GPE* in the beginning, absorbing more knowledge from *GAA*, while in the last few years, *GEN* and *EPF* tend to mutually exchange knowledge with *GAA*, although the weights on the links in both directions become less significant (Fig. 4c). More interestingly, we also find a back-nurture mode as shown in Fig. 4d. Field *GEN* at first absorbs more knowledge from *EOA* than what it provides to *EOA*, but later the situation is inverted. Finally, fields *IPR* and *CM1* shows another pattern, the mutual mode, indicating that they have exchanged knowledge in an almost symmetric way over the whole period. Similar evolution modes have also been seen in the remaining six fields (see SI). These different evolution patterns clearly demonstrate that the processes of knowledge creation and transfer across fields can be highly heterogeneous.

## Discussion

Knowledge sharing and transfer across scientific disciplines, and cross-fertilization are increasingly recognized as crucial factors to breakthrough innovation in science^12,39,40^. The temporal network approach we have proposed in this article can be useful to shed lights on the evolution of knowledge within a field and on the dynamic patterns of influences between different fields. Our study case application has shown that major developments in physics can influence a field for many decades and can even trigger knowledge production in other fields. Indeed, the patterns of cross-fertilization vary greatly among the different disciplines of physics and can also show marked transitions over time. For instance, the physics of gases and plasmas has consistently absorbed knowledge from atomic and molecular physics and from electromagnetism over the last 3 decades. Other fields such as condensed matter and interdisciplinary physics have instead always shared and mutually exchanged knowledge. Finally, we have revealed interesting transitions from absorbing to mutual modes, for instance in the case of the physics of elementary particles, a field of physics that has initially been strongly influenced by astronomy and astrophysics, but in the new century has also contributed to the progress of these latter disciplines. Our findings not only shed new lights on the basic laws governing the development of scientific fields, but can also have practical implications on the future development of economic policies and research strategies.

## Methods

### Data

The data set contains 435, 717 articles published by the American Physical Society (APS) from year 1985 until the end of 2015. Publication date, Physics and Astronomy Classification Scheme (PACS) codes, and bibliography have been extracted for each article. The PACS codes are grouped into a five-level hierarchy and each of them indicates a very specific field of physics. As an example, the PACS code $\textit{64.60.aq}$, indicating the field ”Networks”, belongs to the broader field ”Equations of state, phase equilibria, and phase transitions” (PACS 64) and further belongs to top-level field ”Condensed Matter: Structural, Mechanical and Thermal Properties” (PACS 60). Here, we consider the PACS codes at the highest level, which classify the physics into ten main fields (Table 1). Each article is associated with up to four PACS codes. With regard to the bibliography, only the citations referring to articles published in the APS journals were considered.

### Null model and statistically significant networks

To characterize the flow of knowledge across fields and at different time periods, the statistical significance of each contribution has been validated with respect to an appropriately chosen null model. For each couple of fields all the citations from papers published in citing year *t* to papers published in cited year $t-n$ have been considered. Let ${\mathrm{X}}_{t}^{\mathrm{citing}}$ be the field of citing papers published in year *t*, and ${\mathrm{Y}}_{t-n}^{\mathrm{cited}}$ the field of cited papers published in year $t-n$. We indicate as $P (\alpha _{t-n}, \beta _{t}) = {\mathrm{Pr}}({\mathrm{Y}}_{t-n}^{\mathrm{cited}}=\alpha , {\mathrm{X}}_{t}^{\mathrm{citing}}=\beta )$ the joint probability that papers published in year *t* in field $\beta$ cite papers published in year $t-n$ in field $\alpha$. Such a probability can be written as:

4 $$P(\alpha _{t-n}, \beta _{t})= {\mathrm{Pr}}(\mathrm{Y}_{t-n}^{\mathrm{cited}}=\alpha | {\mathrm{X}}_{t}^{\mathrm{citing}}=\beta ) \times {\mathrm{Pr}}({\mathrm{X}}_{t}^{\mathrm{citing}}=\beta )$$

where ${\mathrm{Pr}}({\mathrm{Y}}_{t-n}^{\mathrm{cited}}=\alpha | {\mathrm{X}}_{t}^{\mathrm{citing}}=\beta )$ is the conditional probability of ${\mathrm{Y}}_{t-n}^{\mathrm{cited}}=\alpha$ given that ${\mathrm{X}}_{t}^{\mathrm{citing}}=\beta$, and ${\mathrm{Pr}}({\mathrm{X}}_{t}^{\mathrm{citing}}=\beta )$ is the marginal probability. We then consider a null model in which the papers published in year *t* in field ${\mathrm{X}}$ randomly select papers published in year $t-n$ as their citations, regardless of which fields they belong to. Hence, the joint probabilities in the null model can be written in terms of the marginal probabilities as:

5 $$\begin{aligned} P^{\mathrm{rand}}(\alpha _{t-n}, \beta _{t})={\mathrm{Pr}}({\mathrm{Y}}_{t-n}^{\mathrm{cited}}=\alpha ) \cdot {\mathrm{Pr}}({\mathrm{X}}_{t}^{\mathrm{citing}}=\beta ) \end{aligned}$$

By calculating the ratio $\phi (\alpha _{t-n}, \beta _{t})$ between the two probabilities in Eqs. (4) and (5):

6 $$\phi (\alpha _{t-n}, \beta _{t})= \frac{ {\mathrm{Pr}}({\mathrm{Y}}_{t-n}^{\mathrm{cited}}=\alpha |{\mathrm{X}}_{t}^{\mathrm{citing}}=\beta )}{{\mathrm{Pr}}({\mathrm{Y}}_{t-n}^{\mathrm{cited}}=\alpha )}$$

we were able to quantify how the observed flows of knowledge deviate from the flows expected to arise simply from random choices. A value $\phi (\alpha _{t-n},\beta _{t})=1$ has been adopted as critical threshold to distinguish whether the knowledge flow from field $\alpha$ to field $\beta$ is statistically significant, with $\phi (\alpha _{t-n},\beta _{t})>1$ indicating that field $\beta$ in year *t* is more likely to have extracted knowledge from field $\alpha$ in year $t-n$ than would be expected at random.

### Network analysis

To characterize the networks of knowledge flow we have evaluated the network reciprocity and we have performed a motif analysis.

1. *Reciprocity*. For each year *t* we have computed the reciprocity coefficient^41^ of the knowledge flow network as:
  7 $$\begin{aligned} \rho _t=\frac{\sum \limits _{\alpha \ne \beta } (w_{\alpha \rightarrow \beta }^{\Delta t' \rightarrow t}-\overline{w}_{\Delta t' \rightarrow t})(w_{\beta \rightarrow \alpha }^{\Delta t' \rightarrow t}-\overline{w}_{\Delta t' \rightarrow t})}{\sum \limits _{\alpha \ne \beta }(w_{\alpha \rightarrow \beta }^{\Delta t' \rightarrow t}-\overline{w}_{\Delta t' \rightarrow t})^2} \end{aligned}$$
  where the average value $\overline{w}_{\Delta t' \rightarrow t} \equiv \sum _{\alpha \ne \beta }w_{\alpha \rightarrow \beta }^{\Delta t' \rightarrow t}/N(N-1)$ indicates the mean of the link weights and $\Delta t'=[1, 5]$. The reciprocity coefficient $\rho _{t}$ ranges from $-1$ to 1, and allows us to distinguish between antireciprocal ($\rho _t<0$) and reciprocal ($\rho _t>0$) networks. We have only considered in the calculation the top half pairs of mutual links with the largest weights.
2. *Motifs*. In this study, we focus on three-node motif analysis^36^. There are 13 different possible connected subgraphs of three nodes. In order to measure the statistical significance of each subgraph *g*, we have computed the *Z*-score $Z_g$ defined as
  8 $$\begin{aligned} Z_{g} = (N_{g}-\langle N^{rand}_{g} \rangle )/\sigma _{g} \end{aligned}$$
  where $N_{g}$ is the number of times subgraph *g* appears in the network, and $\langle N^{rand}_{g} \rangle$ and $\sigma _{g}$ are respectively average and standard deviation of the number of times subgraph *g* occurs in an ensemble of randomized graphs with the same degree distribution as the original network. For each year, we have generated an ensemble of 5000 different randomized samples of the original network using the configuration model.

## Supplementary information

Supplementary information.
