# Supplementary material for: The evolution of knowledge within and across fields in modern physics
Source: Sci Rep. 2020 Jul 21;10:12097. doi: 10.1038/s41598-020-68774-w (PMC7374558; doi:10.1038/s41598-020-68774-w)
Supplement: Supplementary file 1 — Supplementary information. [file 41598_2020_68774_MOESM1_ESM.pdf]

Supplementary Information

The evolution of knowledge within and across fields in modern physics

Ye Sun and Vito Latora

Supplementary Note 1: Evolution of internal knowledge flows for the remaining six fields of physics.

To check whether these patterns are universal, we have further investigated the evolution of internal flows for the remaining six fields of physics. As shown in Fig. S2, we have observed similar evolution characteristics to that of four fields in Fig. 3b-e: fields *ATM*, *GPE* and *CM2* exhibit a similar evolution pattern to field *NUC*, while the evolution characteristics of *GEN* and *EOA* are more similar to that of *EPF* and *GAA*. More specifically, by comparing the change in each row, we find that fields *ATM*, *GPE* and *CM2* show an increasingly high degree of self-reference over time, while, conversely, *GEN* and *EOA* tend to become less and less self-dependent. Focusing on the variation in each column, one can see that fields such as *ATM*, *GPE* and *CM2* show a decreasing trend from most recent times to the past. By contrast, fields *GEN* and *EOA* exhibit a maximum of the values along the anti-diagonal line.

Supplementary Note 2: The process of identifying evolution patterns across fields.

In order to identify the evolution patterns of knowledge flows, we have studied all the evolutionary trajectories of significant knowledge flows across fields. Specifically, for each field, we first select two closely related fields which have the largest average weights of knowledge flows to that field, and then plot their respective evolutionary trajectories in the same way as in Fig. 4b-e. Finally, we summarize all these results into the four major modes as mentioned in the text.

The evolutionary trajectories of four fields (namely *GPE*, *GAA*, *EOA* and *IPR*) have been presented in the text, and that of the remaining six fields have been shown in Fig. S3. One can see that these six fields represent similar evolution patterns, for example, field *NUC* exhibits an "absorbing to mutual" mode (Fig. S3c), where *NUC* has absorbed more knowledge from fields *EPF* and *GAA* in the early stage, then tends to mutually exchange

knowledge with them; field *CM1* shows an "absorbing mode" (Fig. S3e), where *CM1* has been absorbing knowledge from field *CM2* throughout the entire time period.

Supplementary Figures

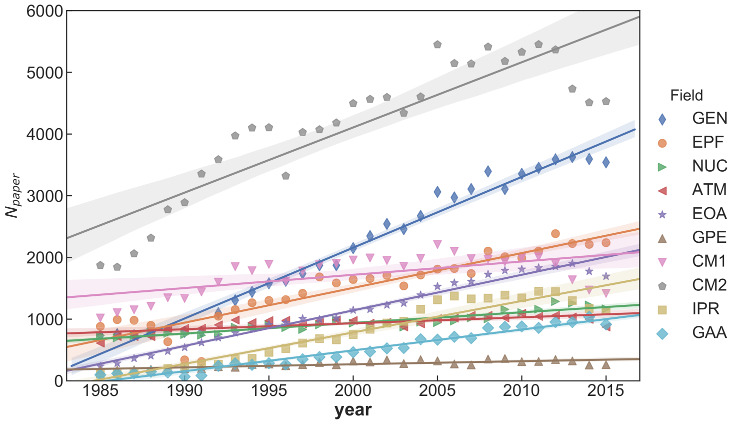

Supplementary Figure S 1: The linear regression line of N_{paper} versus time in each field. The number of publications in each field shows a linearly increase over the last three decades but with different growth rates. ΔN_{paper} is defined as the slop efficient of linear regression model. The solid line and the shaded area represent the regression line and the 95% confidence interval, respectively.

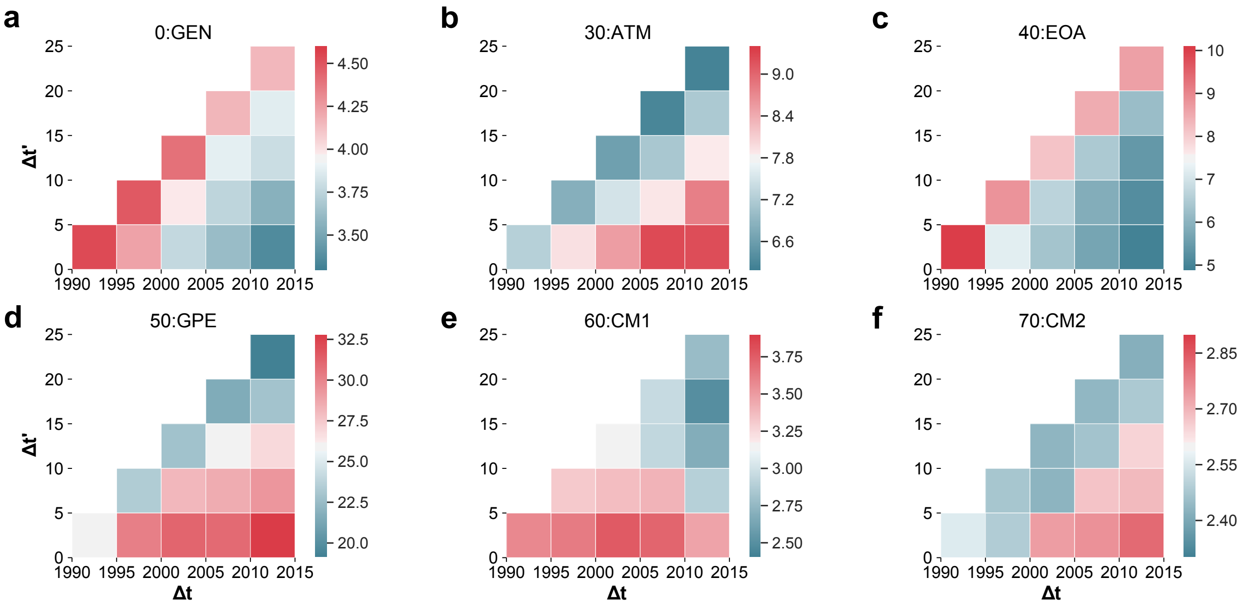

Supplementary Figure S 2: Evolution of internal knowledge flows for the remaining six fields of physics in two-dimensional plots Δt, $\Delta t'$. By comparing the change in each row, we find that fields *ATM*, *GPE* and *CM2* show an increasingly high degree of self-reference over time, while, conversely, *GEN* and *EOA* tend to become less and less self-dependent. Focusing on the variation in each column, we can examine the effect of reference age on the significance of internal knowledge transfer. One can see that fields such as *ATM*, *GPE* and *CM2* show a decreasing trend from most recent times to the past. By contrast, we observe unexpected pattern for *GEN* and *EOA*, where both fields exhibit a maximum of the values along the anti-diagonal line. The lengths of each citing period Δt and cited period $\Delta t'$ are both equal to 5 years.

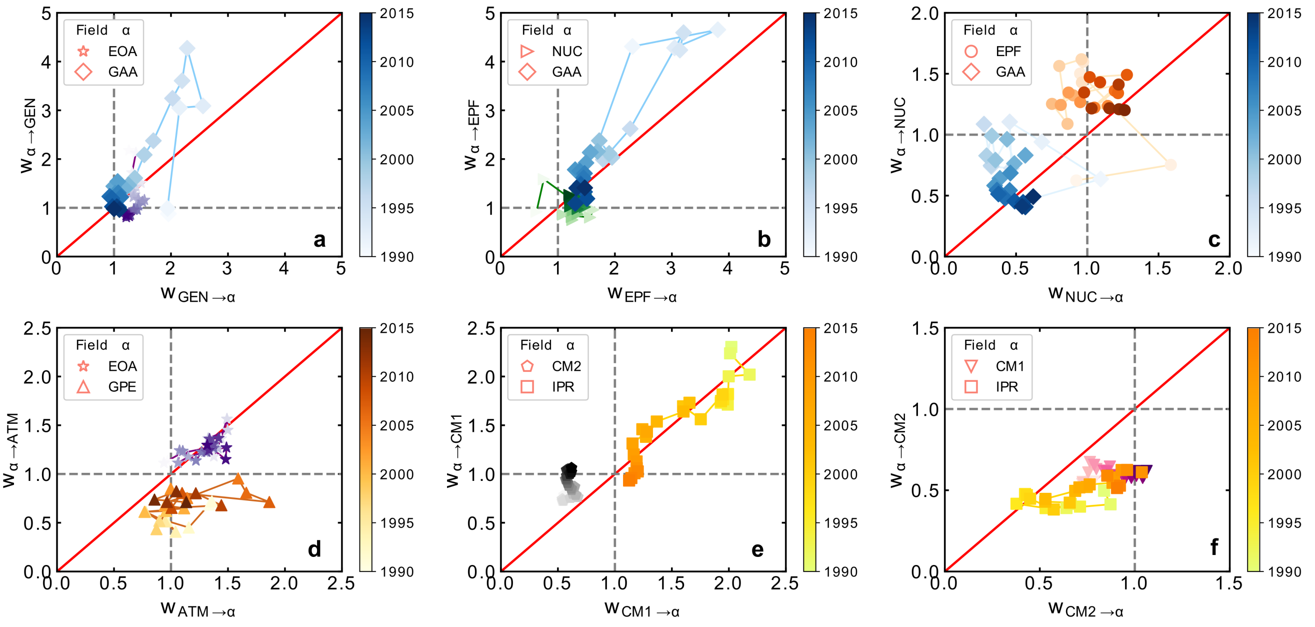

Supplementary Figure S 3: The evolution patterns of knowledge flows in the remaining six fields of physics. For each field, we consider two most relevant fields to observe their evolutionary trajectories. Symbol color (from light to dark) indicates the years from 1990 to 2015, while the lines join consecutive years to help following the trajectories. The bisector red line corresponds to the case of perfectly symmetric knowledge flows between the two fields.
